# Supplementary material for: The genomic basis of the plant island syndrome in Darwin’s giant daisies
Source: Nat Commun. 2022 Jun 28;13:3729. doi: 10.1038/s41467-022-31280-w (PMC9240058; doi:10.1038/s41467-022-31280-w)

Supplementary Information

The genomic basis of the plant island syndrome in Darwin's giant daisies

Cerca et al.

Table of contents

Supplementary Table 01 Flow cytometry estimates **Page 2**

Supplementary Table 02. Repeat and transposable element characterization of the *Scalesia atractyloides* assembly **Page 3**

Supplementary Table 03. Pairwise matrix of conserved orthologous sequences (COS) assignment. **Page 4**

Supplementary Table 04 Significantly enriched GO terms, Cellular components (CC). **Page 6**

Supplementary Table 05. Significantly enriched GO terms, biological functions (BP). **Page 7**

Supplementary Table 06. Significantly enriched GO terms, Biological functions (BP). **Page 9**

Supplementary Table 07. Significantly enriched GO terms, Cellular component (CC). **Page 11**

Supplementary Table 08. Significantly enriched GO terms, Cellular component (CC). **Page 12**

Supplementary Table 09. Significantly enriched GO terms, Molecular Function (MF). **Page 13**

Supplementary Figure 01. IsoSeq Transcriptome data report. **Page 15**

Supplementary Figure 02. Upset plot including five Asteraceae genome assemblies. **Page 16**

Supplementary Figure 03. Hierarchical clustering of selected *k*-mers. **Page 17**

Supplementary Figure 04. K_s plots. **Page 18**

Supplementary Figure 05. Differences in transposable element and repeat content per subgenome. **Page 19**

Supplementary Figure 06. Density distribution of pairwise comparisons of Jukes-Cantor divergence. **Page 20**

Supplementary Figure 07. STRING analysis of *Scalesia* genes under selection. **Page 21**

Supplementary Table 01. Flow Cytometry estimates. Genome size estimates in the studied species including average and standard deviation.

Taxon	1C-Genome size (Gbp)	
	**Mean**	**SD**
Scalesia atractyloides	3.91	0.070
Scalesia baurii ssp. *hopkinsii*	3.99	0.036
Scalesia gordilloi	3.93	0.089
Viguiera tucumanensis	3.83	0.097

Supplementary Table 02. Repeat and transposable element characterization of the *Scalesia atractyloides* assembly. For each repeat or transposable element we include the number of elements, length in base pairs and % of the genome occupied.

Element	Number of elements	Length occupied (bp)	% of sequence
Retroelements	808,501	1,569,493,759	48.79 %
SINEs:	0	0	0 %
Penelope	0	0	0 %
LINEs:	45,417	28,617,548	0.89 %
CRE/SLACS	13,623	6,564,519	0.20 %
L2/CR1/Rex	3,042	4,713,410	0.15 %
R1/LOA/Jockey	0	0	0 %
R2/R4/NeSL	0	0	0 %
RTE/Bov-B	6,128	2,342,838	0.07%
L1/CIN4	22,624	14,996,781	0.47 %
LTR elements:	763,084	1,540,876,211	47.9 %
BEL/Pao	0	0	0 %
Ty1/Copia	276,179	521,239,565	16.2 %
Gypsy/DIRS1	482,535	1,014,580,035	31.54 %
Retroviral	0	0	0 %
DNA transposons	71,490	32,877,488	1.02 %
hobo-Activator	17,945	6,852,805	0.21 %
Tc1-IS630-Pogo	0	0	0 %
En-Spm	0	0	0 %
MuDR-IS905	0	0	0 %
PiggyBac	0	0	0 %
Tourist/Harbinger	30,205	12,698,648	0.39 %
Other (Mirage, P-element, Transib)	0	0	0 %
Rolling-circles	7,490	2,744,511	0.09 %
Unclassified	2,037,841	846,577,345	26.32 %
Total interspersed repeats	-	2,448,948,592	76.13 %
Small RNA	0	0	0 %
Satellites	0	0	0 %
Simple repeats	524	73,608	0 %
Low complexity	0	0	0 %

Supplementary Table 03. Pairwise matrix of conserved orthologous sequences (COS) assignment. We identified duplicate-COS and their genomic location, numbering the number of common COS per chromosome model. For instance, chr10 had a total of 47 COS detected. We detected 39 duplicate COS-pairs shared with chr116, 1 shared with chr27, and another shared with chr7. Green colours show >10 matches, orange shows 3-10 matchees, and yellow shows 1-2 matches.

	1	10	11	116	12	13	14	15	16	1632	1633	1634	17	18	19	2	20	21	22	23	24	25	26	27	28	29	3	30	4	5	6	7	8	9
1	27	-	-	-	-	-	-	-	-	-	-	-	-	-	-	-	-	-	-	-	-	-	-	-	-	-	-	-	-	-	-	-	-	-
10	0	47	-	-	-	-	-	-	-	-	-	-	-	-	-	-	-	-	-	-	-	-	-	-	-	-	-	-	-	-	-	-	-	-
11	0	0	42	-	-	-	-	-	-	-	-	-	-	-	-	-	-	-	-	-	-	-	-	-	-	-	-	-	-	-	-	-	-	-
116	0	39	0	48	-	-	-	-	-	-	-	-	-	-	-	-	-	-	-	-	-	-	-	-	-	-	-	-	-	-	-	-	-	-
12	0	0	0	0	38	-	-	-	-	-	-	-	-	-	-	-	-	-	-	-	-	-	-	-	-	-	-	-	-	-	-	-	-	-
13	0	0	0	0	0	23	-	-	-	-	-	-	-	-	-	-	-	-	-	-	-	-	-	-	-	-	-	-	-	-	-	-	-	-
14	0	0	0	0	0	0	42	-	-	-	-	-	-	-	-	-	-	-	-	-	-	-	-	-	-	-	-	-	-	-	-	-	-	-
15	0	0	0	0	0	15	0	26	-	-	-	-	-	-	-	-	-	-	-	-	-	-	-	-	-	-	-	-	-	-	-	-	-	-
16	14	0	0	0	0	0	1	0	46	-	-	-	-	-	-	-	-	-	-	-	-	-	-	-	-	-	-	-	-	-	-	-	-	-
1632	0	0	0	0	0	0	0	0	0	39	-	-	-	-	-	-	-	-	-	-	-	-	-	-	-	-	-	-	-	-	-	-	-	-
1633	0	0	0	0	0	0	0	0	0	0	21	-	-	-	-	-	-	-	-	-	-	-	-	-	-	-	-	-	-	-	-	-	-	-
1634	8	0	0	0	0	0	0	0	0	1	0	33	-	-	-	-	-	-	-	-	-	-	-	-	-	-	-	-	-	-	-	-	-	-
17	0	0	0	0	0	0	0	0	0	0	0	0	36	-	-	-	-	-	-	-	-	-	-	-	-	-	-	-	-	-	-	-	-	-
18	0	0	0	0	0	0	0	0	0	0	0	0	0	28	-	-	-	-	-	-	-	-	-	-	-	-	-	-	-	-	-	-	-	-
19	0	0	0	0	0	0	0	0	0	0	0	0	0	0	29	-	-	-	-	-	-	-	-	-	-	-	-	-	-	-	-	-	-	-
2	0	0	0	0	0	0	0	0	0	0	0	0	0	0	0	32	-	-	-	-	-	-	-	-	-	-	-	-	-	-	-	-	-	-
20	0	0	0	0	0	0	0	0	0	0	13	0	0	0	0	0	30	-	-	-	-	-	-	-	-	-	-	-	-	-	-	-	-	-
21	0	0	0	0	1	0	0	0	0	0	0	0	0	0	0	0	0	25	-	-	-	-	-	-	-	-	-	-	-	-	-	-	-	-
22	0	0	0	0	0	0	0	0	15	0	0	0	0	0	0	1	0	14	37	-	-	-	-	-	-	-	-	-	-	-	-	-	-	-
23	0	0	0	0	0	0	34	0	0	0	0	0	0	0	0	0	0	0	0	45	-	-	-	-	-	-	-	-	-	-	-	-	-	-
24	0	0	0	0	0	0	0	0	0	0	0	0	0	0	0	0	10	0	0	0	23	-	-	-	-	-	-	-	-	-	-	-	-	-
25	0	0	0	0	33	0	0	0	0	0	0	0	0	0	0	0	0	0	0	0	0	35	-	-	-	-	-	-	-	-	-	-	-	-
26	0	0	0	0	0	0	0	0	0	0	0	0	32	0	0	0	0	0	0	0	0	0	42	-	-	-	-	-	-	-	-	-	-	-

27	0	1	0	0	0	0	0	0	0	0	0	0	0	0	25	0	0	0	0	0	0	0	0	28	-	-	-	-	-	-	-	-	-	-
28	0	0	0	0	0	0	0	0	5	0	0	19	0	0	0	0	0	0	0	0	2	0	0	0	27	-	-	-	-	-	-	-	-	-
29	0	0	0	0	0	0	0	0	0	0	0	0	0	0	0	0	0	0	0	0	0	0	0	0	0	25	-	-	-	-	-	-	-	-
3	0	0	0	0	0	0	0	0	2	0	0	0	0	0	0	0	0	0	1	0	3	0	0	0	0	0	22	-	-	-	-	-	-	-
30	0	0	36	0	0	0	0	0	0	0	0	0	0	0	0	0	0	0	0	0	0	0	0	0	0	0	0	30	-	-	-	-	-	-
4	0	0	0	0	0	0	0	0	1	0	0	0	0	0	0	28	0	0	1	0	0	0	0	0	0	0	0	0	34	-	-	-	-	-
5	0	0	1	0	0	0	0	0	0	0	0	0	0	0	0	0	0	0	0	0	0	0	0	0	0	23	0	0	0	35	-	-	-	-
6	0	0	0	0	0	0	0	0	0	0	0	0	0	0	0	0	0	0	0	0	0	0	0	0	0	0	7	0	0	0	35	-	-	-
7	0	1	0	0	0	1	0	0	0	0	0	0	0	0	1	0	0	6	0	0	0	0	0	0	0	0	0	0	0	0	17	28	-	-
8	0	0	0	0	0	0	0	0	0	0	0	0	0	27	0	0	0	0	0	0	0	0	0	0	0	0	0	0	1	0	0	0	29	-
9	0	0	0	0	0	0	0	0	0	33	0	0	0	0	0	0	0	0	0	0	0	0	0	0	0	0	0	0	0	0	0	0	0	40

Supplementary Table 04 Significantly enriched GO terms, Cellular components (CC). CC-GOs were obtained for the 920 genes under selection. We provide a GO-Id, the term, its annotation, the significance and expected valus and the P-value (elimFisher calculated, one-sided test) for each GO.

ID	Term	Annotated	Significant	Expected	elimFisher
1	GO:0005872	minus-end kinesin complex	96	8	0.0014
2	GO:0072686	mitotic spindle	130	9	0.0026
3	GO:0032153	cell division site	119	8	0.0054
4	GO:0005778	peroxisomal membrane	1185	40	0.0068
5	GO:0031461	cullin-RING ubiquitin ligase complex	585	23	0.0069
6	GO:0000776	kinetochore	399	17	0.0090
7	GO:0005876	spindle microtubule	720	26	0.0121
8	GO:0031519	PcG protein complex	66	5	0.0160
9	GO:0005694	chromosome	2165	69	0.0221
10	GO:0030142	COPI-coated Golgi to ER transport vesicl...	1	1	0.0223
11	GO:0000229	cytoplasmic chromosome	214	10	0.0228
12	GO:0009508	plastid chromosome	214	10	0.0228
13	GO:0000777	condensed chromosome kinetochore	154	8	0.0228
14	GO:0000779	condensed chromosome, centromeric region	154	8	0.0228
15	GO:0035102	PRC1 complex	11	2	0.0240
16	GO:0035861	site of double-strand break	11	2	0.0240
17	GO:0090734	site of DNA damage	11	2	0.0240
18	GO:0005677	chromatin silencing complex	76	5	0.0277
19	GO:0000152	nuclear ubiquitin ligase complex	104	6	0.0294
20	GO:0055028	cortical microtubule	827	27	0.0334
21	GO:0031463	Cul3-RING ubiquitin ligase complex	56	4	0.0364
22	GO:0030981	cortical microtubule cytoskeleton	834	27	0.0364
23	GO:0005881	cytoplasmic microtubule	837	27	0.0378
24	GO:0030863	cortical cytoskeleton	846	27	0.0422
25	GO:0070939	Dsl1/NZR complex	2	1	0.0442
26	GO:1990879	CST complex	2	1	0.0442
27	GO:0009537	proplastid	2	1	0.0442
28	GO:0009528	plastid inner membrane	964	30	0.0443
29	GO:0000151	ubiquitin ligase complex	702	29	0.0452

Supplementary Table 05. Significantly enriched GO terms, biological functions (BP). BP-GOs were obtained for the ***contracted gene families***. We provide a GO-Id, the term, its annotation, the significance and expected values and the P-value (elimFisher calculated, one-sided test) for each GO.

ID	Term	Annotated	Significant	Expected	elimFisher
1	GO:0051865	protein autoubiquitination	222	13	5.8e-20
2	GO:1902006	negative regulation of proline biosynthe...	20	8	9.2e-20
3	GO:1903553	positive regulation of extracellular exo...	6	6	9.8e-19
4	GO:0061635	regulation of protein complex stability	26	8	1.1e-18
5	GO:0071629	cytoplasm protein quality control by the...	47	8	2.3e-16
6	GO:0009414	response to water deprivation	8037	32	1.2e-15
7	GO:1903533	regulation of protein targeting	16	6	7.8e-15
8	GO:0060178	regulation of exocyst localization	25	6	1.7e-13
9	GO:0010600	regulation of auxin biosynthetic process	194	9	4.7e-13
10	GO:0009901	anther dehiscence	662	12	2.3e-12
11	GO:0010966	regulation of phosphate transport	45	6	7.7e-12
12	GO:0090378	seed trichome elongation	202	8	3.8e-11
13	GO:0033591	response to L-ascorbic acid	28	5	1.0e-10
14	GO:0006513	protein monoubiquitination	40	5	6.8e-10
15	GO:0000209	protein polyubiquitination	384	8	6.1e-09
16	GO:0009630	gravitropism	1997	14	6.5e-09
17	GO:0009938	negative regulation of gibberellic acid ...	437	8	1.7e-08
18	GO:0043562	cellular response to nitrogen levels	464	8	2.7e-08
19	GO:0009684	indoleacetic acid biosynthetic process	717	9	4.8e-08
20	GO:0090059	protoxylem development	195	6	6.0e-08
21	GO:0048759	xylem vessel member cell differentiation	203	6	7.6e-08
22	GO:0006887	exocytosis	262	6	3.4e-07
23	GO:0010200	response to chitin	3600	15	1.5e-06
24	GO:2000024	regulation of leaf development	2304	11	1.8e-05
25	GO:0071456	cellular response to hypoxia	5729	17	2.4e-05
26	GO:0030104	water homeostasis	2237	10	8.4e-05
27	GO:0070417	cellular response to cold	727	6	0.00011
28	GO:0036377	arbuscular mycorrhizal association	741	6	0.00013
29	GO:0009787	regulation of abscisic acid-activated si...	4389	17	0.00013
30	GO:0010252	auxin homeostasis	776	6	0.00016
31	GO:0033993	response to lipid	11211	33	0.00019
32	GO:0062034	L-pipecolic acid biosynthetic process	27	2	0.00039
33	GO:1902290	positive regulation of defense response ...	926	6	0.00042
34	GO:0009742	brassinosteroid mediated signaling pathw...	3265	11	0.00042

35	GO:0002238	response to molecule of fungal origin	3299	11	0.00046
36	GO:0033506	glucosinolate biosynthetic process from ...	32	2	0.00055
37	GO:0006511	ubiquitin-dependent protein catabolic pr...	1382	14	0.00063
38	GO:1901701	cellular response to oxygen-containing c...	10052	32	0.00072
39	GO:0009789	positive regulation of abscisic acid-act...	2943	10	0.00078
40	GO:0001173	DNA-templated transcriptional start site...	1	1	0.00108
41	GO:0043392	negative regulation of DNA binding	1	1	0.00108
42	GO:2000280	regulation of root development	3667	11	0.00114
43	GO:0009735	response to cytokinin	3272	10	0.00178
44	GO:2000070	regulation of response to water deprivat...	3366	10	0.00220
45	GO:0050832	defense response to fungus	7460	16	0.00235
46	GO:0046621	negative regulation of organ growth	72	2	0.00274
47	GO:0047484	regulation of response to osmotic stress	3551	10	0.00328
48	GO:0048731	system development	16694	33	0.00338
49	GO:0090333	regulation of stomatal closure	2424	8	0.00355
50	GO:0009610	response to symbiotic fungus	1985	7	0.00460
51	GO:0009609	response to symbiotic bacterium	1529	6	0.00534
52	GO:0009725	response to hormone	14346	33	0.00556
53	GO:0048831	regulation of shoot system development	6119	19	0.00657
54	GO:2000652	regulation of secondary cell wall biogen...	2202	7	0.00805
55	GO:1900426	positive regulation of defense response ...	2270	7	0.00945
56	GO:1901420	negative regulation of response to alcoh...	3023	8	0.01314
57	GO:1905958	negative regulation of cellular response...	3023	8	0.01314
58	GO:0009788	negative regulation of abscisic acid-act...	3023	8	0.01314
59	GO:0002237	response to molecule of bacterial origin	3156	8	0.01675
60	GO:0043068	positive regulation of programmed cell d...	931	4	0.01725
61	GO:0071310	cellular response to organic substance	11202	31	0.01734
62	GO:0042742	defense response to bacterium	8510	18	0.01738
63	GO:0009737	response to abscisic acid	9526	24	0.02823
64	GO:0097305	response to alcohol	9617	24	0.03087
65	GO:0009617	response to bacterium	9246	18	0.03209
66	GO:0071396	cellular response to lipid	7800	29	0.03449
67	GO:1900150	regulation of defense response to fungus	2955	7	0.03531
68	GO:0052542	defense response by callose deposition	3706	8	0.03962
69	GO:0042631	cellular response to water deprivation	2495	6	0.04801

Supplementary Table 06. Significantly enriched GO terms, Biological functions (BP). BP-GOs were obtained for the ***expanded gene families***. We provide a GO-Id, the term, its annotation, the significance and expected values and the P-value (elimFisher calculated, one-sided test) for each GO.

ID	Term	Annotated	Significant	Expected	elimFisher
1	GO:0033486	delphinidin 3-O-glucoside biosynthetic p...	33	33	< 1e-30
2	GO:0050766	positive regulation of phagocytosis	29	29	< 1e-30
3	GO:0010466	negative regulation of peptidase activit...	83	57	< 1e-30
4	GO:0030187	melatonin biosynthetic process	72	34	< 1e-30
5	GO:0010951	negative regulation of endopeptidase act...	55	29	< 1e-30
6	GO:0015976	carbon utilization	60	29	< 1e-30
7	GO:1901847	nicotinate metabolic process	87	30	< 1e-30
8	GO:0006730	one-carbon metabolic process	92	30	< 1e-30
9	GO:0009710	tropane alkaloid biosynthetic process	126	32	< 1e-30
10	GO:0009701	isoflavonoid phytoalexin biosynthetic pr...	58	25	< 1e-30
11	GO:0006020	inositol metabolic process	166	33	< 1e-30
12	GO:0002103	endonucleolytic cleavage of tetracistron...	16	14	8.9e-27
13	GO:0030497	fatty acid elongation	159	28	1.1e-26
14	GO:0033485	cyanidin 3-O-glucoside biosynthetic proc...	302	33	2.3e-24
15	GO:0009807	lignan biosynthetic process	127	24	8.2e-24
16	GO:0051851	modulation by host of symbiont process	90	21	4.7e-23
17	GO:0000290	deadenylation-dependent decapping of nuc...	21	12	2.2e-19
18	GO:0032259	methylation	2243	68	1.2e-16
19	GO:0000305	response to oxygen radical	402	29	1.3e-16
20	GO:0098869	cellular oxidant detoxification	405	29	1.6e-16
21	GO:0080024	indolebutyric acid metabolic process	366	27	9.0e-16
22	GO:0043473	pigmentation	655	33	3.4e-14
23	GO:0006694	steroid biosynthetic process	981	40	5.8e-14
24	GO:0009772	photosynthetic electron transport in pho...	65	13	8.1e-14
25	GO:0006666	3-keto-sphinganine metabolic process	21	9	2.4e-13
26	GO:0051555	flavonol biosynthetic process	766	34	4.9e-13
27	GO:0009708	benzyl isoquinoline alkaloid biosyntheti...	658	31	1.2e-12
28	GO:0080088	spermidine hydroxycinnamate conjugate bi...	742	32	5.3e-12
29	GO:1990110	callus formation	713	29	2.2e-10
30	GO:0000028	ribosomal small subunit assembly	205	16	3.2e-10
31	GO:0009820	alkaloid metabolic process	1887	95	4.0e-10
32	GO:0080026	response to indolebutyric acid	647	27	5.4e-10
33	GO:0009805	coumarin biosynthetic process	848	31	6.8e-10
34	GO:0010597	green leaf volatile biosynthetic process	975	30	6.3e-08

35	GO:0009759	indole glucosinolate biosynthetic proces...	1003	30	1.2e-07
36	GO:0033962	P-body assembly	169	12	1.5e-07
37	GO:0031640	killing of cells of other organism	294	15	3.3e-07
38	GO:0016554	cytidine to uridine editing	261	14	4.5e-07
39	GO:0030148	sphingolipid biosynthetic process	105	9	1.2e-06
40	GO:0051289	protein homotetramerization	338	15	1.9e-06
41	GO:0008033	tRNA processing	217	12	2.2e-06
42	GO:0009809	lignin biosynthetic process	3911	68	3.4e-06
43	GO:1900871	chloroplast mRNA modification	68	7	5.4e-06
44	GO:0080156	mitochondrial mRNA modification	323	14	5.5e-06
45	GO:0006561	proline biosynthetic process	383	15	8.7e-06
46	GO:0010197	polar nucleus fusion	360	14	1.9e-05
47	GO:0071244	cellular response to carbon dioxide	1284	29	4.3e-05
48	GO:0006397	mRNA processing	1932	38	5.7e-05
49	GO:0050830	defense response to Gram-positive bacter...	73	6	9.3e-05
50	GO:0036377	arbuscular mycorrhizal association	741	19	0.00020
51	GO:0042542	response to hydrogen peroxide	3539	56	0.00036
52	GO:0031525	menthol biosynthetic process	94	6	0.00037
53	GO:0010115	regulation of abscisic acid biosynthetic...	537	15	0.00038
54	GO:1900865	chloroplast RNA modification	207	14	0.00046
55	GO:0006885	regulation of pH	1617	29	0.00184
56	GO:0006760	folic acid-containing compound metabolic...	131	6	0.00210
57	GO:0006333	chromatin assembly or disassembly	781	16	0.00577
58	GO:0010304	PSII associated light-harvesting complex...	173	6	0.00812
59	GO:0046677	response to antibiotic	83	4	0.00973
60	GO:0009750	response to fructose	875	15	0.03132
61	GO:0002213	defense response to insect	1781	26	0.03530
1	GO:0033486	delphinidin 3-O-glucoside biosynthetic p...	33	33	< 1e-30
2	GO:0050766	positive regulation of phagocytosis	29	29	< 1e-30
3	GO:0010466	negative regulation of peptidase activit...	83	57	< 1e-30
4	GO:0030187	melatonin biosynthetic process	72	34	< 1e-30
5	GO:0010951	negative regulation of endopeptidase act...	55	29	< 1e-30
6	GO:0015976	carbon utilization	60	29	< 1e-30
7	GO:1901847	nicotinate metabolic process	87	30	< 1e-30
8	GO:0006730	one-carbon metabolic process	92	30	< 1e-30

Supplementary Table 07. Significantly enriched GO terms, Cellular component (CC). CC-GOs were obtained for the ***contracted gene families***. We provide a GO-Id, the term, its annotation, the significance and expected values and the P-value (elimFisher calculated, one-sided test) for each GO.

ID	Term	Annotated	Significant	Expected	elimFisher
1	GO:0045335	phagocytic vesicle	6	6	1.1e-18
2	GO:0000145	exocyst	31	6	7.6e-13
3	GO:0070062	extracellular exosome	32	6	9.4e-13
4	GO:0009501	amyloplast	345	8	2.9e-09
5	GO:0085042	periarbuscular membrane	292	6	6.9e-07
6	GO:0000306	extrinsic component of vacuolar membrane	30	3	4.7e-06
7	GO:0000139	Golgi membrane	2435	12	5.5e-06
8	GO:0032586	protein storage vacuole membrane	353	5	3.8e-05
9	GO:0016021	integral component of membrane	11678	24	0.0001
10	GO:0005618	cell wall	8035	16	0.0044
11	GO:0009524	phragmoplast	1638	6	0.0079
12	GO:0005789	endoplasmic reticulum membrane	6329	13	0.0123
13	GO:0009535	chloroplast thylakoid membrane	2665	7	0.0227
14	GO:0005654	nucleoplasm	4943	10	0.0337

Supplementary Table 08. Significantly enriched GO terms, Cellular component (CC). CC-GOs were obtained for the ***expanded gene families***. We provide a GO-Id, the term, its annotation, the significance and expected values and the P-value (elimFisher calculated, one-sided test) for each GO.

ID	Term	Annotated	Significant	Expected	elimFisher
1	GO:0012511	monolayer-surrounded lipid storage body	69	29	< 1e-30
2	GO:0042597	periplasmic space	13	13	8.2e-27
3	GO:0033115	cyanelle thylakoid membrane	224	22	1.3e-15
4	GO:0070111	organellar chromatophore	260	21	6.1e-12
5	GO:0009539	photosystem II reaction center	45	10	2.1e-11
6	GO:0005763	mitochondrial small ribosomal subunit	142	13	2.3e-09
7	GO:0009535	chloroplast thylakoid membrane	2665	59	6.8e-09
8	GO:0009523	photosystem II	213	23	1.2e-08
9	GO:0033095	aleurone grain	176	11	1.9e-06
10	GO:0043245	extraorganismal space	66	7	4.5e-06
11	GO:0005811	lipid droplet	347	39	0.00027
12	GO:0009842	cyanelle	909	39	0.00031
13	GO:0000322	storage vacuole	325	11	0.00049
14	GO:0005615	extracellular space	961	21	0.00079
15	GO:0000785	chromatin	641	16	0.00085
16	GO:0005835	fatty acid synthase complex	8	2	0.00270
17	GO:0010287	plastoglobule	527	13	0.00281
18	GO:0070118	organellar chromatophore thylakoid membr...	101	5	0.00357
19	GO:0005576	extracellular region	9339	127	0.00561
20	GO:0000932	P-body	564	12	0.01228
21	GO:0009579	thylakoid	3760	75	0.03675

Supplementary Table 09. Significantly enriched GO terms, Molecular Function (MF). MF-GOs were obtained for the ***contracted gene families***. We provide a GO-Id, the term, its annotation, the significance and expected values and the P-value (elimFisher calculated, one-sided test) for each GO.

ID	Term	Annotated	Significant	Expected	elimFisher
1	GO:0047434	indolepyruvate decarboxylase activity	15	9	6.3e-24
2	GO:0103075	indole-3-pyruvate monooxygenase activity	16	9	1.4e-23
3	GO:0004499	N,N-dimethylaniline monooxygenase activi...	23	9	1.0e-21
4	GO:0061630	ubiquitin protein ligase activity	581	14	1.4e-15
5	GO:0051787	misfolded protein binding	170	8	1.8e-11
6	GO:0050660	flavin adenine dinucleotide binding	380	9	3.9e-10
7	GO:0050661	NADP binding	450	9	1.7e-09
8	GO:0080102	3-methylthiopropyl glucosinolate S-oxyge...	17	4	3.6e-09
9	GO:0080103	4-methylthiopropyl glucosinolate S-oxyge...	17	4	3.6e-09
10	GO:0080104	5-methylthiopropyl glucosinolate S-oxyge...	17	4	3.6e-09
11	GO:0080105	6-methylthiopropyl glucosinolate S-oxyge...	17	4	3.6e-09
12	GO:0080106	7-methylthiopropyl glucosinolate S-oxyge...	17	4	3.6e-09
13	GO:0080107	8-methylthiopropyl glucosinolate S-oxyge...	17	4	3.6e-09
14	GO:0062047	pipecolic acid N-hydroxylase	12	2	8.6e-05
15	GO:0031543	peptidyl-proline dioxygenase activity	30	2	0.00056
16	GO:0043621	protein self-association	3085	10	0.00209
17	GO:0031624	ubiquitin conjugating enzyme binding	59	2	0.00216
18	GO:0030247	polysaccharide binding	1883	7	0.00532
19	GO:0031625	ubiquitin protein ligase binding	1990	6	0.02581
20	GO:0005199	structural constituent of cell wall	252	2	0.03469

Supplementary Table 10. Significantly enriched GO terms, Molecular Function (MF). MF-GOs were obtained for the ***expanded gene families***. We provide a GO-Id, the term, its annotation, the significance and expected values and the P-value (elimFisher calculated, one-sided test) for each GO.

ID	Term	Annotated	Significant	Expected	elimFisher
1	GO:0047434	indolepyruvate decarboxylase activity	15	9	6.3e-24
2	GO:0103075	indole-3-pyruvate monooxygenase activity	16	9	1.4e-23
3	GO:0004499	N,N-dimethylaniline monooxygenase activi...	23	9	1.0e-21
4	GO:0061630	ubiquitin protein ligase activity	581	14	1.4e-15
5	GO:0051787	misfolded protein binding	170	8	1.8e-11
6	GO:0050660	flavin adenine dinucleotide binding	380	9	3.9e-10

7	GO:0050661	NADP binding	450	9	1.7e-09
8	GO:0080102	3-methylthiopropyl glucosinolate S-oxyge...	17	4	3.6e-09
9	GO:0080103	4-methylthiopropyl glucosinolate S-oxyge...	17	4	3.6e-09
10	GO:0080104	5-methylthiopropyl glucosinolate S-oxyge...	17	4	3.6e-09
11	GO:0080105	6-methylthiopropyl glucosinolate S-oxyge...	17	4	3.6e-09
12	GO:0080106	7-methylthiopropyl glucosinolate S-oxyge...	17	4	3.6e-09
13	GO:0080107	8-methylthiopropyl glucosinolate S-oxyge...	17	4	3.6e-09
14	GO:0062047	pipecolic acid N-hydroxylase	12	2	8.6e-05
15	GO:0031543	peptidyl-proline dioxygenase activity	30	2	0.00056
16	GO:0043621	protein self-association	3085	10	0.00209
17	GO:0031624	ubiquitin conjugating enzyme binding	59	2	0.00216
18	GO:0030247	polysaccharide binding	1883	7	0.00532
19	GO:0031625	ubiquitin protein ligase binding	1990	6	0.02581
20	GO:0005199	structural constituent of cell wall	252	2	0.03469

Supplementary Figure 01. IsoSeq Transcriptome data report. A) % of isoforms per gene, showing different categories labeled by colours. B) % of transcripts which are mono or multi-exon; C) Count of all Isoseq transcript length; D) Cunt of multi and mono-exon transcripts.

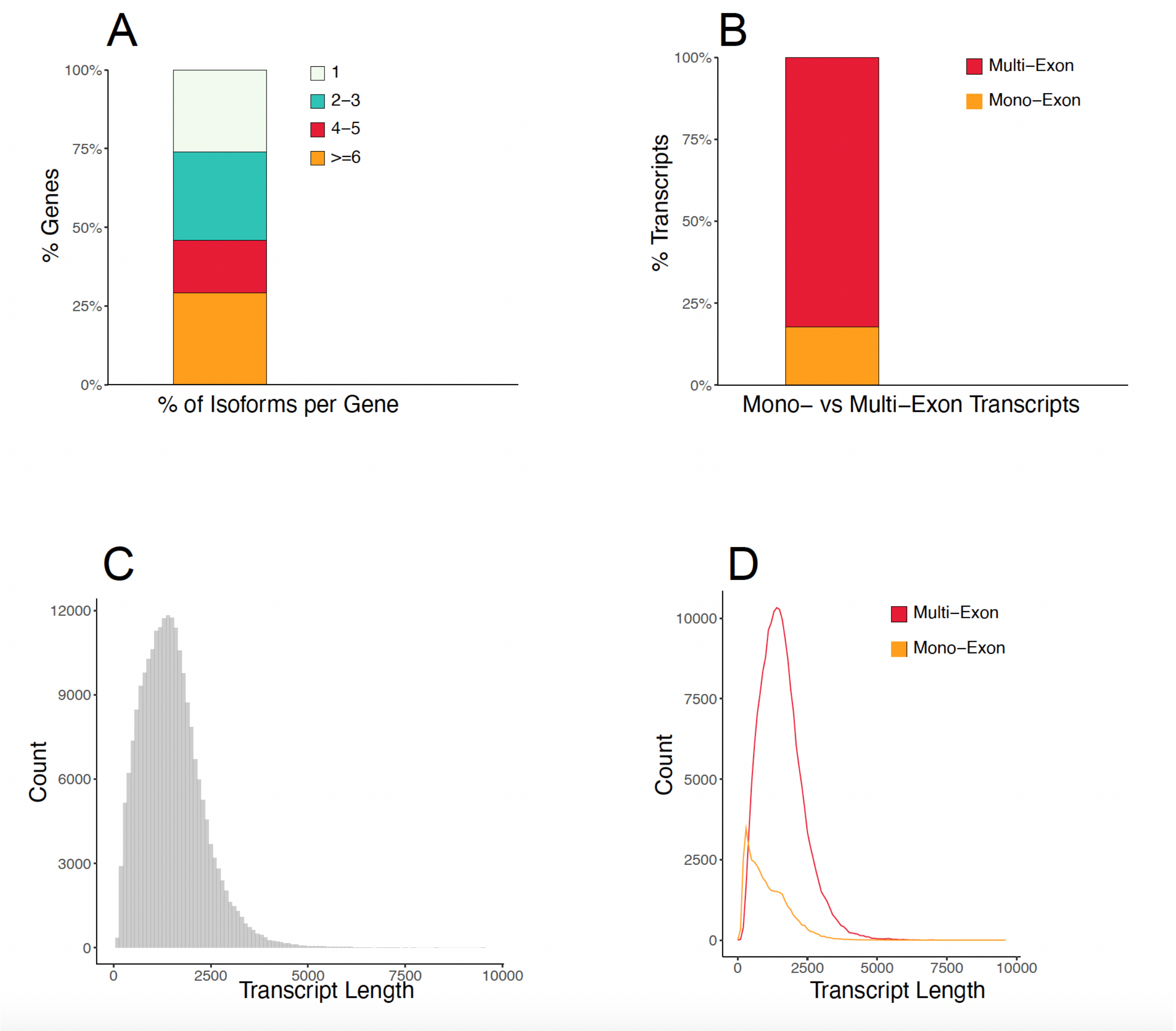

Supplementary Figure 02. Upset plot including five Asteraceae genome assemblies. For each genome we present the number of orthogroups which include genomes from different combinations. For instance, there are 9,191 orthogroups including the five genomes.

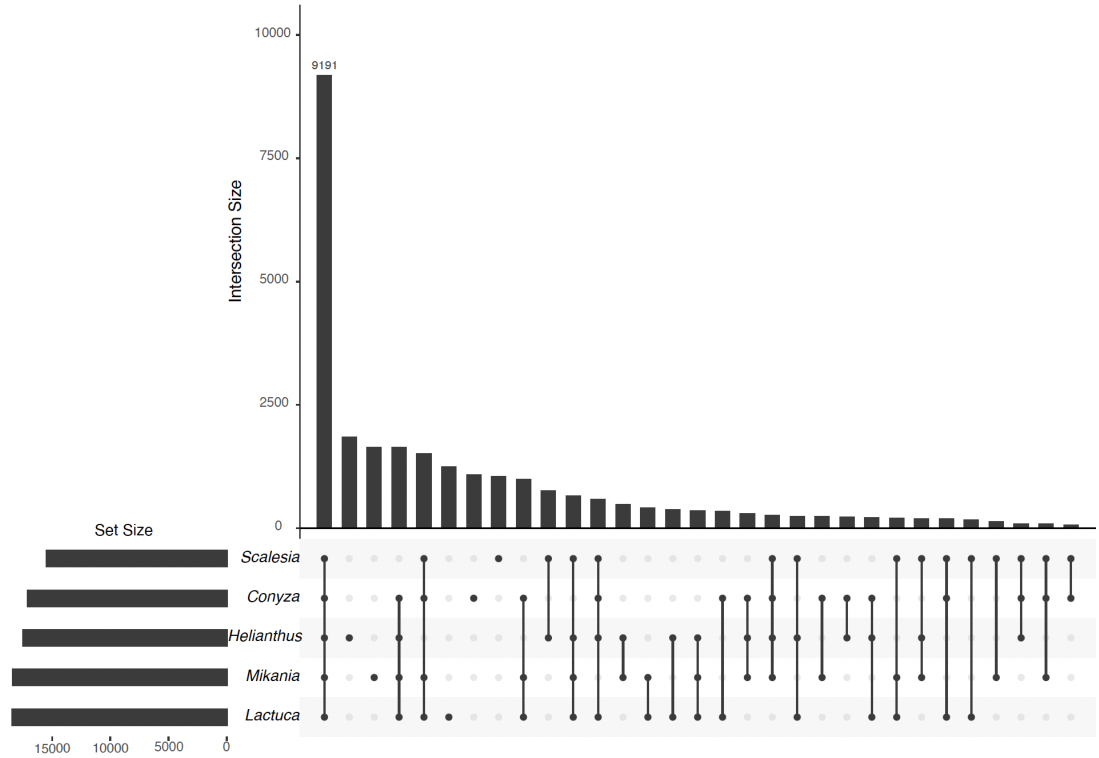

Supplementary Figure 03. Hierarchical clustering of selected *k*-mers. A selection of 13-mers present in high abundance (>100) in the *Scalesia atractyloides* genome assembly, and differentially represented in homologous chromosomes. Hierarchical clustering separated the subgenomes.

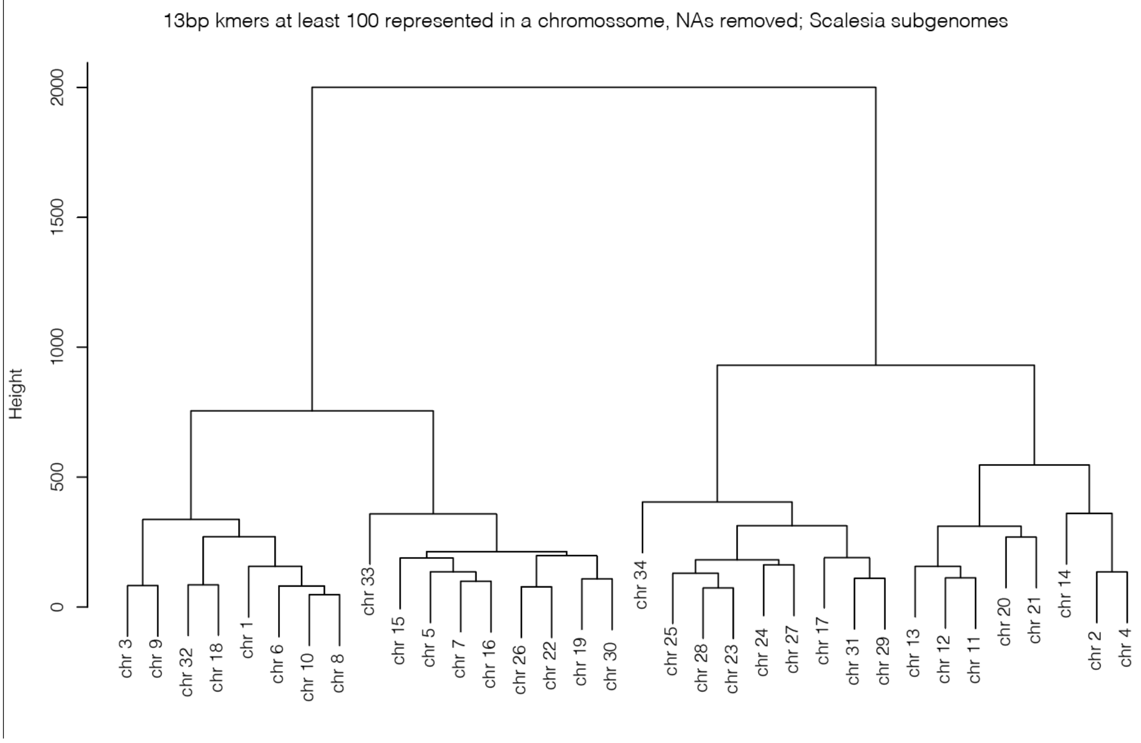

Supplementary Figure 04. K_s plots. Ks plots for *Scalesia* subgenome A vs subgenome B (top), *Scalesia* subgenome A vs *Helianthus* (middle), and *Scalesia* subgenome B vs *Helianthus* (bottom). For each comparison, we report the peak of the distribution for each component.

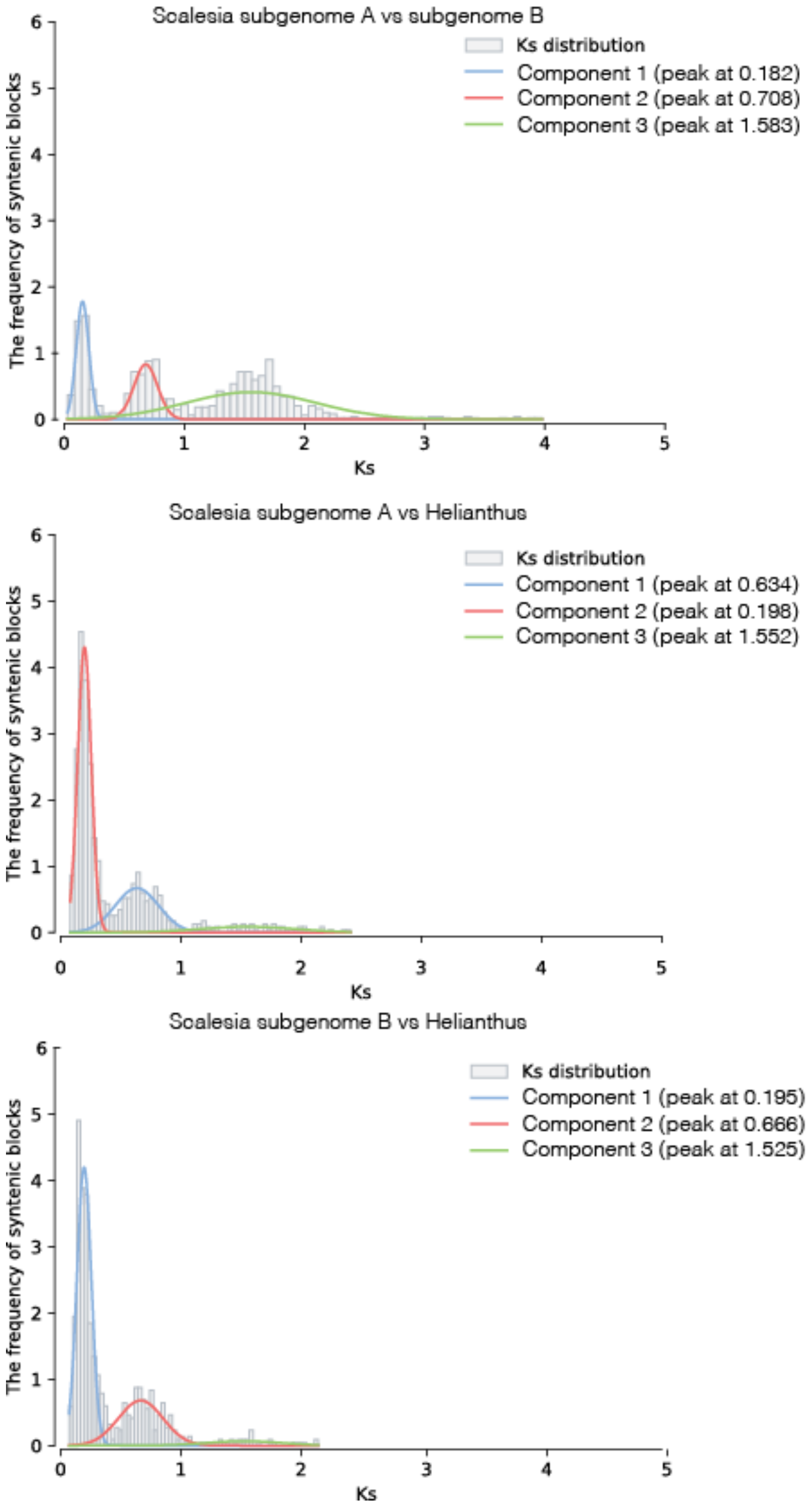

Supplementary Figure 05. Differences in transposable element and repeat content per subgenome. For each family identified by RepeatMasker (*x*-axis), we plot the difference between transposable elements for TE-number differences between subgenomes, for each TE-family.

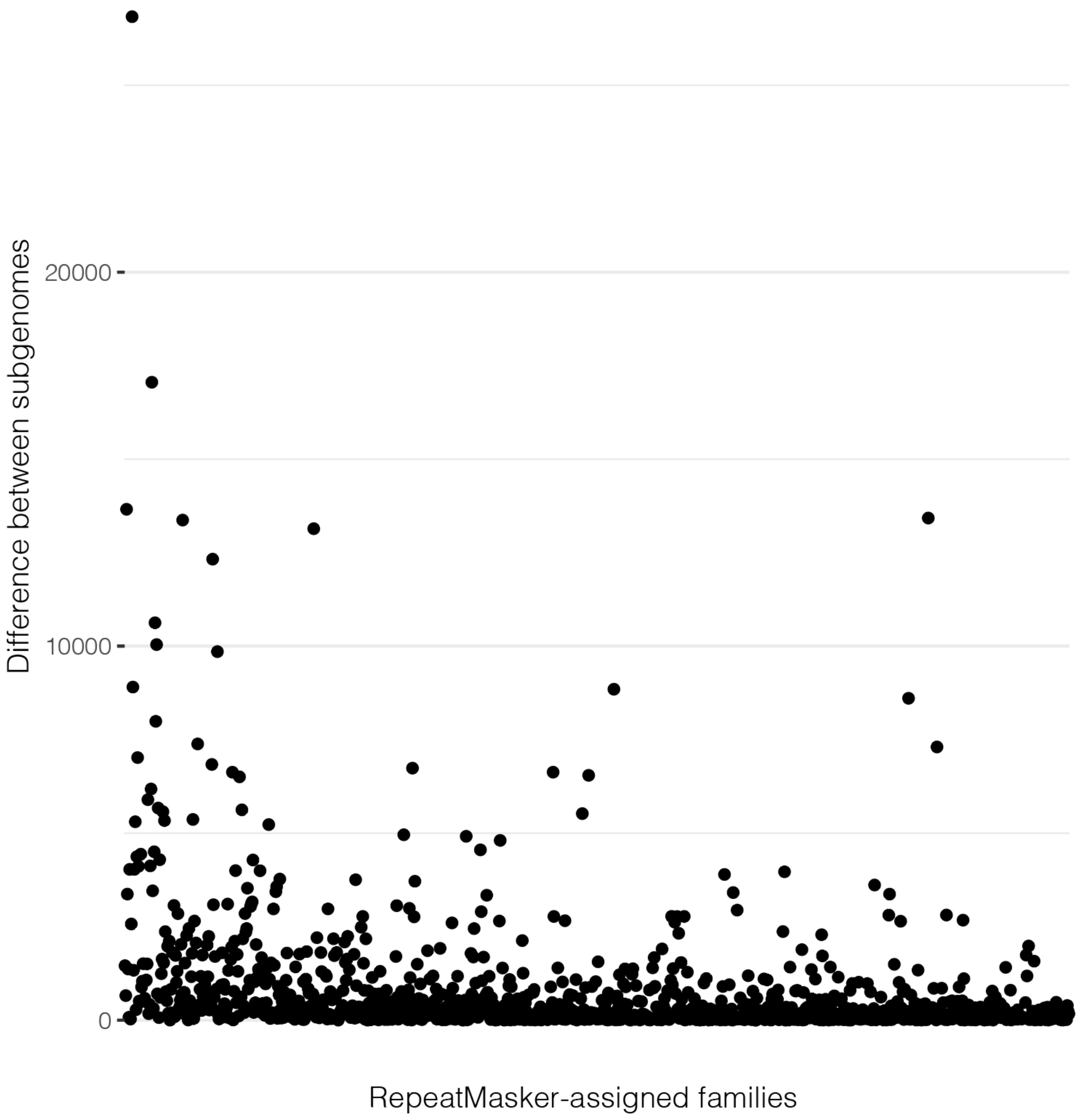

Supplementary Figure 06. Density distribution of pairwise comparisons of Jukes-Cantor divergence. We selected LTR-families evenly represented on the subgenomes, and present in the *Helianthus* genome for this analysis. These families were cleaned by removing missing data and cleaning the sequences using Gblocks. We ended up with 5 high-quality LTR-repeat families, which were used for dating the polyploidization event. In yellow, we plot *Scalesia* vs *Scalesia* LTRs, and in grey we plot *Scalesia* vs *Helianthus* LTRs. The arrows point to the peak of the distribution, which was used to estimate the divergence between *Scalesia* subgenomes.

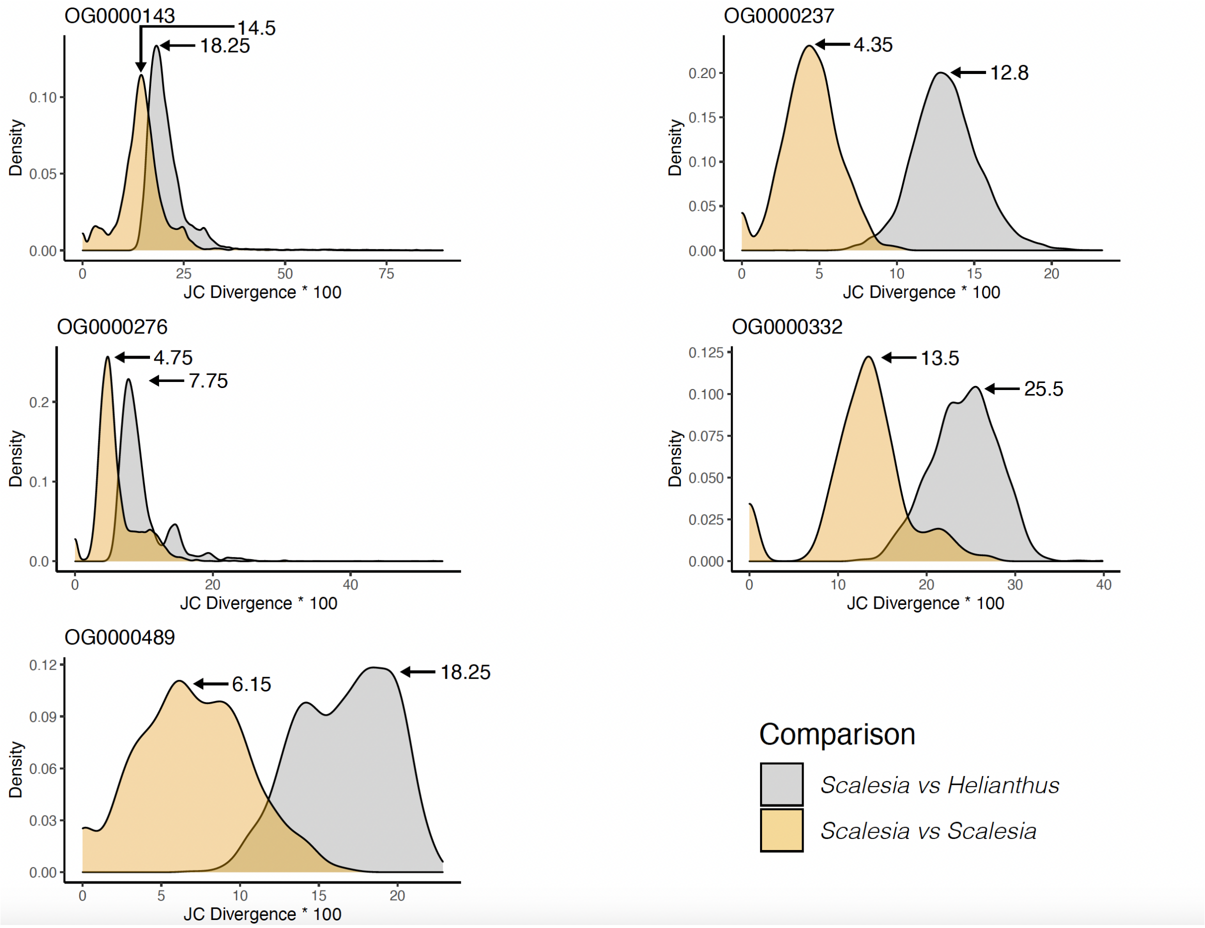

Supplementary Figure 07. STRING analysis of *Scalesia* genes under selection. To explore the potential protein-protein interactions among the *Scalesia* genes under selection, we performed a STRING analysis with the 632 closest Arabidopsis orthologs. Interaction scores of edges were calculated based on the parameters: Experiments, Co-expression, Neighborhood, Gene fusion and Co-occurrence. Edges with interaction score higher than 0.400 were kept in the network. After excluding genes with no physical connection, the STRING network had 627 nodes with 470 edges (PPI enrichment p-value <0.001). To simplify the densely connected network into potential biologically functional clusters, we used the distance matrix obtained from the STRING global scores as the input to perform a k-Means clustering analysis (number of clusters = 6). 4 out of the 6 clusters are enriched for biological processes related GO terms. Cluster 1 (red bubbles) were enriched for the GO term metabolic processes, cluster 3 (lime green bubbles) for histone modifications and chromosome organization, cluster 4 (green bubbles) for response to light, and cluster 6 (purple bubbles) for ribosomal large subunit biogenesis and RNA-processing.

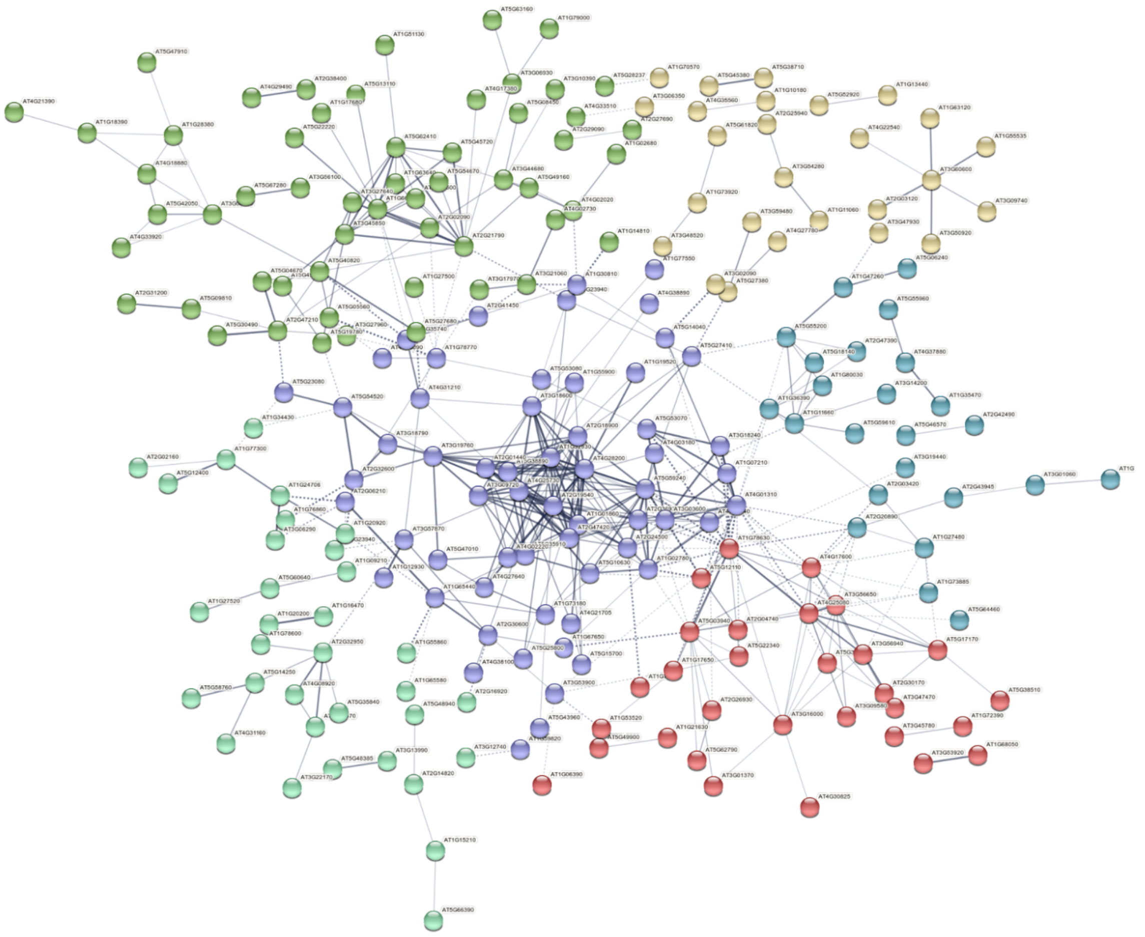

Supplement: Supplementary file 1 — Supplementary Information [file 41467_2022_31280_MOESM1_ESM.pdf]
