# Supplementary material for: The genomic basis of the plant island syndrome in Darwin’s giant daisies
Source: Nat Commun. 2022 Jun 28;13:3729. doi: 10.1038/s41467-022-31280-w (PMC9240058; doi:10.1038/s41467-022-31280-w)
Supplement: Supplementary file 3 — Description of Additional Supplementary Files [file 41467_2022_31280_MOESM3_ESM.pdf]

Description of Additional Supplementary Files

File Name: Supplementary Data 1
Description: Transposable element family distribution per subgenome. Family ID is detailed as output from RepeatMasker.

File Name: Supplementary Data 2
Description: Significantly enriched GO terms, Biological Processes (BP). BP-GOs were obtained for the 920 genes under selection. We provide a GO-Id, the term, its annotation, the significance and expected values and the P-value (elimFisher calculated, one-sided test) for each GO.

File Name: Supplementary Data 3
Description: Significantly enriched GO terms, Molecular functions (MF). MF-GOs were obtained for the 920 genes under selection. We provide a GO-Id, the term, its annotation, the significance and expected valus and the P-value (elimFisher calculated, one-sided test) for each GO.

File Name: Supplementary Data 4
Description: Arabidopsis literature search. We randomly selected 100 genes under selection in Scalesia and, for each of these, we provide an arbitrary orthogroup ID (ID), the closest Arabidopsis ortholog detected (obtained via BLAST; Gene name), the protein name on the Arabidopsis.org database (Protein name), a classification based on function after the literature (broad category), a specific category that describes what was found based on knock-outs and differential gene expression experiments (specific category), specific findings highlighting the results from Arabidopsis papers (specific finders) and the papers where the specific findings, and categories were obtained from (Paper 1-5).
