# Supplementary material for: The genomic basis of the plant island syndrome in Darwin’s giant daisies
Source: Nat Commun. 2022 Jun 28;13:3729. doi: 10.1038/s41467-022-31280-w (PMC9240058; doi:10.1038/s41467-022-31280-w)
Supplement: Supplementary file 5 — Reporting Summary [file 41467_2022_31280_MOESM5_ESM.pdf]

nature portfolio

Corresponding author(s): José Cerca, Michael D. Martin

Last updated by author(s): Dec 8, 2021

Reporting Summary

Nature Portfolio wishes to improve the reproducibility of the work that we publish. This form provides structure for consistency and transparency in reporting. For further information on Nature Portfolio policies, see our Editorial Policies and the Editorial Policy Checklist.

Statistics

For all statistical analyses, confirm that the following items are present in the figure legend, table legend, main text, or Methods section.

n/a	Confirmed	
☐	☒	The exact sample size (*n*) for each experimental group/condition, given as a discrete number and unit of measurement
☒	☐	A statement on whether measurements were taken from distinct samples or whether the same sample was measured repeatedly
☐	☒	The statistical test(s) used AND whether they are one- or two-sided<br>*Only common tests should be described solely by name; describe more complex techniques in the Methods section.*
☒	☐	A description of all covariates tested
☐	☒	A description of any assumptions or corrections, such as tests of normality and adjustment for multiple comparisons
☐	☒	A full description of the statistical parameters including central tendency (e.g. means) or other basic estimates (e.g. regression coefficient) AND variation (e.g. standard deviation) or associated estimates of uncertainty (e.g. confidence intervals)
☐	☒	For null hypothesis testing, the test statistic (e.g. *F*, *t*, *r*) with confidence intervals, effect sizes, degrees of freedom and *P* value noted<br>*Give P values as exact values whenever suitable.*
☒	☐	For Bayesian analysis, information on the choice of priors and Markov chain Monte Carlo settings
☒	☐	For hierarchical and complex designs, identification of the appropriate level for tests and full reporting of outcomes
☒	☐	Estimates of effect sizes (e.g. Cohen's *d*, Pearson's *r*), indicating how they were calculated

Our web collection on statistics for biologists contains articles on many of the points above.

Software and code

Policy information about availability of computer code

Data collection	No software used.
Data analysis	Genome assembly - wtdbg2<br>Genome decontamination - Blobtools v1.1.1<br>Duplicates - purge_dups v1.1.2<br>Scaffolding - HiRise v1<br>Genome assessment - Assemblathon2, BUSCO viridiplantae odb v10<br>Repeat identification - RepeatMasker v4.1.1, RepeatModeler v2.0.1<br>RNA data processing - lima v2.0.0, Isoseq3 refine v3.4.0, Isoseq3 cluster v3.4.0, pbmm2 align v1.4.0, SQANTI3 v1.0.0<br>Gene annotation - MAKER2 pipeline v2.31.9<br>Re-sequencing data processing and demography - FastQC v0.11.8, AdapterRemoval v2.3.1, Trimmomatic v0.39, bwa mem 0.7.17, PSMC v0.6.5<br>COS processing - phyluce v1.6.8<br>k-mer processing - Jellyfish v0.8.9<br>Circos plots - circos v0.69.8<br>Orthology - Blast v2.12, OrthoFinder v2.5.4<br>Selection and gene expansions - CAFE v4.2.1, hyphy v2.5.3.<br>TopGO 2.46.0

For manuscripts utilizing custom algorithms or software that are central to the research but not yet described in published literature, software must be made available to editors and reviewers. We strongly encourage code deposition in a community repository (e.g. GitHub). See the Nature Portfolio guidelines for submitting code & software for further information.

Data

Policy information about availability of data

All manuscripts must include a data availability statement. This statement should provide the following information, where applicable:

- Accession codes, unique identifiers, or web links for publicly available datasets
- A description of any restrictions on data availability
- For clinical datasets or third party data, please ensure that the statement adheres to our policy

The raw data generated in this study have been deposited in the ENA database under accession code https://www.ebi.ac.uk/ena/browser/view/PRJEB52418 . The assembly and the annotation files are available here https://datadryad.org/stash/dataset/doi:10.5061/dryad.8gtht76rh .

Field-specific reporting

Please select the one below that is the best fit for your research. If you are not sure, read the appropriate sections before making your selection.

☐ Life sciences ☐ Behavioural & social sciences ☒ Ecological, evolutionary & environmental sciences

For a reference copy of the document with all sections, see nature.com/documents/nr-reporting-summary-flat.pdf

Ecological, evolutionary & environmental sciences study design

All studies must disclose on these points even when the disclosure is negative.

Study description	We sequenced a genome, using long reads and contiguity-ligation methods. Using this assembly we estimated natural selection, demography, gene-family evolution and subgenome estimation.
Research sample	We sequenced a genome from a plant and obtained RNA from the same individual for reproducibility. We added some closely related species for demographic analyses. Scalesia atrctyloides was selected because it is critically endangered and has low genomic heterozygosity suitable for de-novo assembly.
Sampling strategy	Not applicable
Data collection	DNA was obtained from leaves, RNA from various tissues (flower, leaf early development, leaf late development, root, stem)
Timing and spatial scale	Not applicable
Data exclusions	No data was removed
Reproducibility	The code is available in https://github.com/jcerca/Papers/tree/main/scalesia_genome , and has been double-checked for reproducibility.
Randomization	Not applicable for genome data.
Blinding	Not applicable for genome data

Did the study involve field work? ☐ Yes ☒ No

Reporting for specific materials, systems and methods

We require information from authors about some types of materials, experimental systems and methods used in many studies. Here, indicate whether each material, system or method listed is relevant to your study. If you are not sure if a list item applies to your research, read the appropriate section before selecting a response.

Materials & experimental systems

n/a	Involved in the study
☒	☐ Antibodies
☒	☐ Eukaryotic cell lines
☒	☐ Palaeontology and archaeology
☒	☐ Animals and other organisms
☒	☐ Human research participants
☒	☐ Clinical data
☒	☐ Dual use research of concern

Methods

n/a	Involved in the study
☒	☐ ChIP-seq
☒	☐ Flow cytometry
☒	☐ MRI-based neuroimaging
